# Exposure to urban green spaces and mental health during the COVID-19 pandemic: evidence from two low and lower-middle-income countries

**DOI:** 10.3389/fpubh.2024.1334425

**Published:** 2024-03-01

**Authors:** Muhammad Mainuddin Patwary, Mondira Bardhan, Hüseyin Ertan İnan, Matthew H. E. M. Browning, Asma Safia Disha, Md. Zahidul Haque, Mai Helmy, Sadia Ashraf, Angel M. Dzhambov, Faysal Kabir Shuvo, Md. Ashraful Alam, Sharif Mutasim Billah, Md. Pervez Kabir, Md. Riad Hossain, Md. Golam Azam, Md. Mijanur Rahman, Sarya Swed, Ranjit Sah, Juan J. Montenegro-Idrogo, D. Katterine Bonilla-Aldana, Alfonso J. Rodriguez-Morales

**Affiliations:** ^1^Environment and Sustainability Research Initiative, Khulna, Bangladesh; ^2^Environmental Science Discipline, Life Science School, Khulna University, Khulna, Bangladesh; ^3^Department of Parks, Recreation, and Tourism Management, Clemson University, Clemson, SC, United States; ^4^Department of Tourism Management, Faculty of Tourism, Ondokuz Mayıs University, Samsun, Türkiye; ^5^Department of Environmental Science and Management, North South University, Dhaka, Bangladesh; ^6^Psychology Department, College of Education, Sultan Qaboos University, Muscat, Oman; ^7^Psychology Department, Faculty of Arts, Menoufia University, Shibin el Kom, Egypt; ^8^Department of Hygiene, Faculty of Public Health, Medical University of Plovdiv, Plovdiv, Bulgaria; ^9^Research Group “Health and Quality of Life in a Green and Sustainable Environment”, SRIPD, Medical University of Plovdiv, Plovdiv, Bulgaria; ^10^Environmental Health Division, Research Institute at Medical University of Plovdiv, Medical University of Plovdiv, Plovdiv, Bulgaria; ^11^Institute of Highway Engineering and Transport Planning, Graz University of Technology, Graz, Austria; ^12^Swinburne University of Technology, Hawthorn, VIC, Australia; ^13^Department of Computational Diagnostic Radiology and Preventive Medicine, The University of Tokyo Hospital, Tokyo, Japan; ^14^Department of Civil Engineering, University of Ottawa, Ottawa, ON, Canada; ^15^Institute of Disaster Management, Khulna University Engineering & Technology, Khulna, Bangladesh; ^16^Remote Sensing, Center for Environmental and Geographic Information Services (CEGIS), Dhaka, Bangladesh; ^17^Department of Geography and Environment, Jagannath University, Dhaka, Bangladesh; ^18^Faculty of Medicine, Aleppo University, Aleppo, Syria; ^19^Green City Hospital, Kathmandu, Nepal; ^20^Department of Microbiology, Dr. D. Y. Patil Medical College, Hospital and Research Centre, Dr. D. Y. Patil Vidyapeeth, Pune, India; ^21^Faculty of Health Sciences, Universidad Cientifica del Sur, Lima, Peru; ^22^Infectious and Tropical Diseases Service, Hospital Nacional Dos de Mayo, Lima, Peru; ^23^Research Unit, Universidad Continental, Huancayo, Peru; ^24^Grupo de Investigación Biomedicina, Faculty of Medicine, Fundación Universitaria Autónoma de las Américas-Institución Universitaria Visión de las Américas, Pereira, Colombia; ^25^Gilbert and Rose-Marie Chagoury School of Medicine, Lebanese American University, Beirut, Lebanon

**Keywords:** green space exposure, NDVI, mental health, LMIC, COVID-19

## Abstract

**Introduction:**

The COVID-19 pandemic has had a significant impact on mental health globally, with limited access to mental health care affecting low- and middle-income countries (LMICs) the most. In response, alternative strategies to support mental health have been necessary, with access to green spaces being a potential solution. While studies have highlighted the role of green spaces in promoting mental health during pandemic lockdowns, few studies have focused on the role of green spaces in mental health recovery after lockdowns. This study investigated changes in green space access and associations with mental health recovery in Bangladesh and Egypt across the pandemic.

**Methods:**

An online survey was conducted between January and April 2021 after the first lockdown was lifted in Bangladesh (n = 556) and Egypt (n = 660). We evaluated indoor and outdoor greenery, including the number of household plants, window views, and duration of outdoor visits. The quantity of greenness was estimated using the normalized difference vegetation index (NDVI). This index was estimated using satellite images with a resolution of 10x10m during the survey period (January-April 2021) with Sentinel-2 satellite in the Google Earth Engine platform. We calculated averages within 250m, 300m, 500m and 1000m buffers of the survey check-in locations using ArcGIS 10.3. Multiple linear regression models were used to evaluate relationships between changes in natural exposure and changes in mental health.

**Results:**

The results showed that mental health improved in both countries after the lockdown period. People in both countries increased their time spent outdoors in green spaces after the lockdown period, and these increases in time outdoors were associated with improved mental health. Unexpectedly, changes in the number of indoor plants after the lockdown period were associated with contrasting mental health outcomes; more plants translated to increased anxiety and decreased depression. Refocusing lives after the pandemic on areas other than maintaining indoor plants may assist with worrying and feeling panicked. Still, indoor plants may assist with depressive symptoms for people remaining isolated.

**Conclusion:**

These findings have important implications for policymakers and urban planners in LMICs, highlighting the need to increase access to natural environments in urban areas to improve mental health and well-being in public health emergencies.

## 1 Introduction

The COVID-19 pandemic originated in Wuhan, China, in December 2019 and rapidly became a global crisis. On March 11, 2020, the World Health Organization declared it a pandemic due to its rapid transmission (1). By March 15, 2023, it had infected over 681 million people and resulted in 6.8 million deaths worldwide (2). To contain the viral spread, governments implemented various measures such as lockdowns, curfews, quarantines, and other non-pharmaceutical interventions (3). The Bangladesh government imposed a complete lockdown on March 26, 2020 and continued till May 31, 2020 (4), followed by zonal lockdowns from June 20, 2020 to July 9, 2020 (5). Similarly, Egypt implemented a nationwide lockdown from March 25 to June 27, 2020 (6). With no vaccine and limited understanding of the virus, these forms of social distancing were considered the most effective method of prevention and regulation during some periods of the pandemic (3, 7). However, such restrictions had profound consequences, affecting daily life, economies, and health, leaving many uncertain about the pandemic’s duration and the prospect of gaining control over it (8).

Correspondingly, the pandemic had substantial negative impacts on mental health worldwide, with numerous studies highlighting the harmful effects of social isolation and lockdowns on mental health (9–14). The pandemic led to increased symptoms of depression, anxiety, post-traumatic stress disorder (PTSD), distress, and insomnia (15). One review concluded that over 95% of people reported PTSD symptoms, 72% reported distress, 45% reported anxiety symptoms, and 34% reported insomnia during the pandemic (16). Such impacts were particularly acute in low- and middle-income countries (LMICs) due to their limited access to mental health care (17). A meta-analysis of 40 developing countries reported that distress (29%) and depression (27%) were the most prevalent mental health symptoms during COVID-19 (18).

Research on exposure to green spaces and benefits to mental health has gained attention from researchers and healthcare professionals in recent years. *The stress reduction theory* suggests that green spaces can induce a sense of emotional well-being and a calming effect on individuals (19). Thus, exposure to green spaces can promote relaxation and stress reduction. Further, *attention restoration* could be another established theory that suggests attention restoration in green space is associated with improved psychological well-being, including reduced symptoms of depression and anxiety (19). Earlier studies have shown that exposure to green spaces can have positive impacts on mental health, reducing symptoms of anxiety and depression, as well as promoting overall well-being (20–22). Green spaces have also played an essential role in alleviating the negative mental health burden during the COVID-19 lockdown (23, 24). A study in Spain reported individuals turned to green spaces as a source of comfort, both directly and indirectly, to alleviate the negative effects of the pandemic (25). A study in Bulgaria showed that visible access to the greenery around the home and neighborhood was associated with decreased symptoms of depression and anxiety during the pandemic (26). Window views of greenery have been found to provide micro-restorative episodes that aid in healing, psychological regeneration, and rehabilitation from traumatic events (27), including during lockdowns (26). A study from Italy reported greener views and access to private green spaces were associated with better mental health outcomes (28).

While the mental health benefits of urban green spaces are well understood in high-income countries (HICs) (29–31), the evidence does not represent the diversity of urban living conditions in rapidly urbanizing LMICs. The existing evidence largely excludes the types of urban environments where most of the world’s population lives (32). Consequently, researchers and policymakers should avoid assuming that findings from HICs can be automatically applied to LMICs, given the diverse urban conditions and environmental and cultural differences between these countries (33). Informal settlements and slums often characterize cities in LMICs, and people living in these areas may not have the same level of access to green space as those in more affluent areas (32). This lack of access to green space in low-income cities may have different impacts on mental health than in HIC cities, where the availability and quality of green space may differ. To date, few studies have focused on the association between green space exposure and mental health in LMICs (34). A spatial epidemiological study conducted in Bangladesh (a tropical climate) found a negative correlation between vegetation and psychological well-being (35). A study in India (a sub-tropical climate) found a positive association between lack of park access and depression (36).

Furthermore, a study in Egypt (a hot and dry climate) focused on the impact of the green space on people’s happiness (37). This existing literature shows contrasting findings before the pandemic and does not inform the role of green spaces in mental health during the pandemic. We know only one study that answers this literature gap: spending time in a home garden was associated with less anxiety and stress during the COVID-19 lockdown in India (38).

Further limiting our understanding of green space exposure and mental health during the pandemic is the limited research comparing associations during and after lockdowns. The importance of green spaces during lockdowns has been widely recognized (26–28). The green space’s role in mental health recovery after lockdowns may be equally important. As the world continues to navigate through the pandemic’s aftermath, policymakers and mental health professionals must focus on developing effective interventions and policies to support mental health recovery. The focus of the current study is to examine exposure to green spaces and mental health recovery following lockdowns in two LMICs. The two countries represent radically different climates, with Bangladesh being tropical and Egypt being deserts (39). This distinction allows for exploring how access to nature and its impact on mental health may differ in different climate zones, which is essential in developing targeted interventions for LMICs. Our primary research question (RQ) included: How were changes in nature access associated with recovery from poor mental health after lockdowns? By emphasizing the recovery phase of the pandemic, we sought to complement existing research, which primarily focused on the immediate effects of the pandemic (23, 40).

## 2 Materials and methods

### 2.1 Study design

A retrospective study design was adopted to conduct the study. Online surveys were administered between January and April 2021, when lockdowns were lifted and people were getting used to the ‘new normal’ of the pandemic in Bangladesh and Egypt. The survey used a free version of an online survey platform, KoBoToolbox,^1^ allowing the survey to be distributed and completed without face-to-face interaction. The target population was the general people aged 18 and above. We used a snowball sampling approach and distributed the invitations through email and various social media platforms (e.g., Facebook, WhatsApp, LinkedIn). Participants were informed about the study and allowed to withdraw at any time. A total of 1,216 respondents (556 from Bangladesh and 660 from Egypt) were included in the analysis.

The survey consisted of six sections. These included socio-demographic information, potential risk factors, potential mediators, perceived exposure to indoor and outdoor nature, and self-reported mental health. Respondents were asked to provide data for two time points: the period of lockdown during the pandemic and the post-lockdown period (the current time of the survey administration). The survey also collected the geolocation of the participants, which was used to calculate objective greenness levels at both periods. The survey form was written in English and translated into local languages. The research was approved by the ethics committee of the Institute of Disaster Management, Khulna University of Engineering and Technology, Khulna, Bangladesh and the Psychology Department, Faculty of Arts, Menoufia University, Egypt.

### 2.2 Nature exposure measures

#### 2.2.1 Perceptions

We adopted two measures for perceived indoor nature exposure: the number of household plants and window views (41). The first was measured by asking, “How many indoor plants are in this home?” Respondents answered it numerically (i.e., 0, 1, or 2 plants). The second was measured by asking about the visibility of 13 built or natural spaces/elements through any of the windows of their residence. Respondents scored these as present or absent, providing binary measures for each type. The spaces/elements included industrial building(s), courtyard/housing block patio, urban area (houses and streets), road, park(s), river(s), lake(s), agricultural area(s), countryside, woodland(s)/forest, hill(s)/mountain(s), and little access to outdoor visual elements because of neighbor’s walls or no window views. Responses were categorized as natural (park, river, lake, agriculture, countryside, woodland/forest, hill/mountain), built (industrial buildings, houses and street, road, neighbors wall), or mixed (at least one item in the natural category and at least one in the built category). Respondents indicated their perceived indoor nature exposure during the lockdown and at the current time.

We measured perceived outdoor nature exposure with two items: spaces accessed outdoors and hours spent outdoors. Spaces accessed outdoors were asked first and included responses for during the lockdown (“Which outdoor spaces did you have physical access to during the COVID-19 lockdown?”) and current time (“Which outdoor green spaces have you visited in the last four weeks?”). Time spent outdoors was also asked for these two-time points and was measured by asking, ‘How many hours did you spend each day at the places you visited, on average?’ If participants spent time at two or more outdoor spaces, they were requested to provide the total average number of hours per day for all those spaces. Duration was scored on a 4-point scale: <1 h, 1–2 h, 3–5 h, and > 5 h, following a previous study (42). Because of slight differences in how the survey was administered between countries, we could not use the data from the spaces accessed outdoors in analyses. Our perceived outdoor nature exposure was limited to total time outdoors during the lockdown and current time.

#### 2.2.2 Objective greenness

To measure the quantity of greenness, we initially considered three metrics: the normalized difference vegetation index (NDVI), enhanced vegetation index (EVI), and soil-adjusted vegetation index (SAVI). NDVI is a remote sensing index used to estimate vegetation cover and productivity. It is based on the difference in reflectance between the near-infrared (NIR) and red (RED) bands of electromagnetic radiation. The result of the NDVI calculation ranges from −1 to 1, with higher values indicating more excellent vegetation cover and productivity (43). EVI is similar to NDVI but was designed to reduce the influences of atmospheric and soil noise on the vegetation signal. We considered EVI since it can be an appropriate measure in regions with high atmospheric aerosol content, such as deserts, which may influence NDVI. SAVI corrects for the influence of soil brightness on the vegetation signal and is also particularly useful in regions with high soil brightness, such as deserts and semi-arid regions (44). These indices were estimated using satellite images with a resolution of 10 × 10m during the survey period (January–April 2021) with Sentinel-2 satellite in the Google Earth Engine platform. We calculated averages within 250 m, 300 m, 500 m and 1,000 m buffers of the survey check-in locations using ArcGIS 10.3 (Esri, Redlands, CA, United States). NDVI and EVI values were highly correlated, *r* = 0.68 to 1.0 (Supplementary Figure S1), and there were no changes between the lockdown and the current time in SAVI. For these reasons, NDVI was chosen for analysis. NDVI values across buffer sizes were also highly correlated *r* = 0.88 to 0.96 (Supplementary Figure S1), and 500 m was selected to correspond to the previous nature and health research (45).

### 2.3 Mental health measures

Several scales are generally accepted to measure mental health, including the 7-item Generalized Anxiety Disorder (GAD-7) scale, 9-item Patient Health Questionnaire (PHQ-9), and 4-item Patient Health Questionnaire (PHQ-4) to evaluate mental health (46–48). To keep the questionnaire brief, we used the 2-item Patient Health Questionnaire (PHQ-2) and 2-item General Anxiety Disorder (GAD-2) to measure depression and anxiety disorder, respectively. GAD-2 evaluated the frequency of participants feeling nervous, anxious, or on edge and unable to stop or control worrying over the last two weeks in lockdown and the current period (49). PHQ-2 rated the frequency of feeling down, depressed, or hopeless and having little interest or pleasure in doing things over the past two weeks for the same period (50). The response options were on 4-point Likert scales: 0 (not at all), 1 (several days), 2 (more than half of the day), and 3 (almost every day). The total score ranges from 0 to 6, with a cut-off value of ≥3 indicating a higher risk of anxiety and depression.

### 2.4 Covariates

We assessed socio-demographic variables, potential risk factors, and household characteristics that might affect mental health outcomes. Based on previous research (51, 52), we asked about gender, age, marital status, and family income in local currency (BDT and Egyptian pound) converted to USD (0–100, 101–200, 201–400, and > 400 USD). We also evaluated past COVID-19 diagnoses of respondents and their family members, providing four options: tested positive at least once, tested negative and never tested further positive, never tested, or at least one of the family members tested positive for COVID-19. We asked whether respondents had a chronic physical illness since these can influence mental health (53) and green space use (54). Two potential risk factors were also measured, including habit of smoking and body mass index (BMI). Smoking can have negative impacts on mental health (27), and BMI can influence quality of life (55).

### 2.5 Analysis

In this study, both the exposure and outcome variables were assessed at two-time points, allowing us to capture changes and developments over time in response to the intervention or exposure. In contrast, covariates, including socio-demographic and health-related variables, were collected at a single point in time. This design was chosen to examine how changes in exposure levels corresponded to changes in the outcome variables and whether socio-demographic characteristics influenced these changes. We compared the lockdown and current time values for all variables. These were tested with chi-square values of independence for count variables and paired sample *t-*tests for continuous variables. Next, we compared change scores for all variables between the countries using chi-squared tests and independent sample *t*-tests. Change scores were calculated as the current time value minus the lockdown period value.

Multiple linear regression models were used to assess associations between changes in mental health and changes in natural exposure. Separate models were run for anxiety and depression in each country. Bangladesh models included socio-demographic variables, potential risk factors, household characteristics, COVID-19 diagnosis, changes in the number of indoor plants, changes in window views of nature, changes in time spent outdoors, and greenness (NDVI-500). Models showed no evidence of multicollinearity, as demonstrated by variance inflation factor (VIF) values ≤2.5 and pairwise correlation values ≤0.7 (Supplementary Table S2). Normality tests on outcome variables were performed using Skewness and Kurtosis with a critical value of 3.0 and a visual inspection of histograms (Supplementary Table S3). The outcome values of depression and anxiety were distributed normally. All analyses were conducted with SPSS v21 (IBM, Armonk, New York) with an alpha set for significance at *p* < 0.05.

## 3 Results

### 3.1 Descriptive statistics

The characteristics of respondents are provided in Table 1. Most were female, aged >25 years, single, lived in an urban area, and reported a monthly family income between 201 and 400 USD. Most also reported they never tested for COVID-19 and did not have a long-standing illness or habit of smoking. Egypt respondents were more likely to be female, over 25, single, urban residents, and earning monthly incomes of 201–400 USD. The proportion of respondents who had never been tested for COVID-19 was higher in Bangladesh than in Egypt. In comparison, the proportion of respondents without a long-term illness or habit of smoking was higher in Egypt than in Bangladesh.

**Table 1:** Descriptive statistics of respondents in Bangladesh and Egypt (*N* = 1,216).

	Total	Bangladesh (*N* = 556)		Egypt (*N* = 660)		Country differences
	N (%) or Mean(SD)	N	%	*N*	%	*p*
	1,216 (100)	556	100	660	100	
Gender						0.000
Male	472 (38.8)	280	50.4	192	29.1	
Female	744 (61.2)	276	49.6	468	70.9	
Age						0.000
≤25	257(21.1)	252	45.3	5	0.8	
>25	959(78.7)	304	54.7	655	99.2	
Marital status						0.000
Single	1,108 (91.1)	451	81.1	657	99.5	
Married	108 (8.9)	105	18.9	3	0.5	
Current place of residence						0.000
Urban	1,026 (84.4)	434	78.1	592	89.7	
Rural	190 (15.6)	122	21.9	68	10.3	
Monthly family income (USD)						0.000
0–100	167 (13.7)	142	25.5	25	3.8	
101–200	189 (15.5)	118	21.2	71	10.8	
201–400	732 (60.2)	168	30.2	564	85.5	
>400	128 (10.5)	128	23	0	0	
COVID-19 diagnosis						0.004
Tested positive at least once	62 (5.1)	23	4.1	39	5.9	
Never tested positive	137 (11.3)	68	12.2	69	10.5	
Never tested	323 (75.9)	437	78.6	486	73.6	
At least one family member has tested positive	94 (7.7)	28	5	66	10	
Presence of long-standing illness						0.030
Yes	88 (7.2)	50	9	38	5.8	
No	1,128 (92.8)	506	91	622	94.2	
Habit of smoking						0.000
Yes	97 (8)	82	14.7	15	2.3	
No	1,119 (92)	474	85.3	645	97.7	
BMI	23.66 (4.1)	23.53	4.14	23.77	4.07	0.307

Comparisons between countries calculated with independent sample t-tests or chi-squared tests depending on measure (count vs. continuous).

### 3.2 Changes in mental health and nature exposure

**Figure 1:**
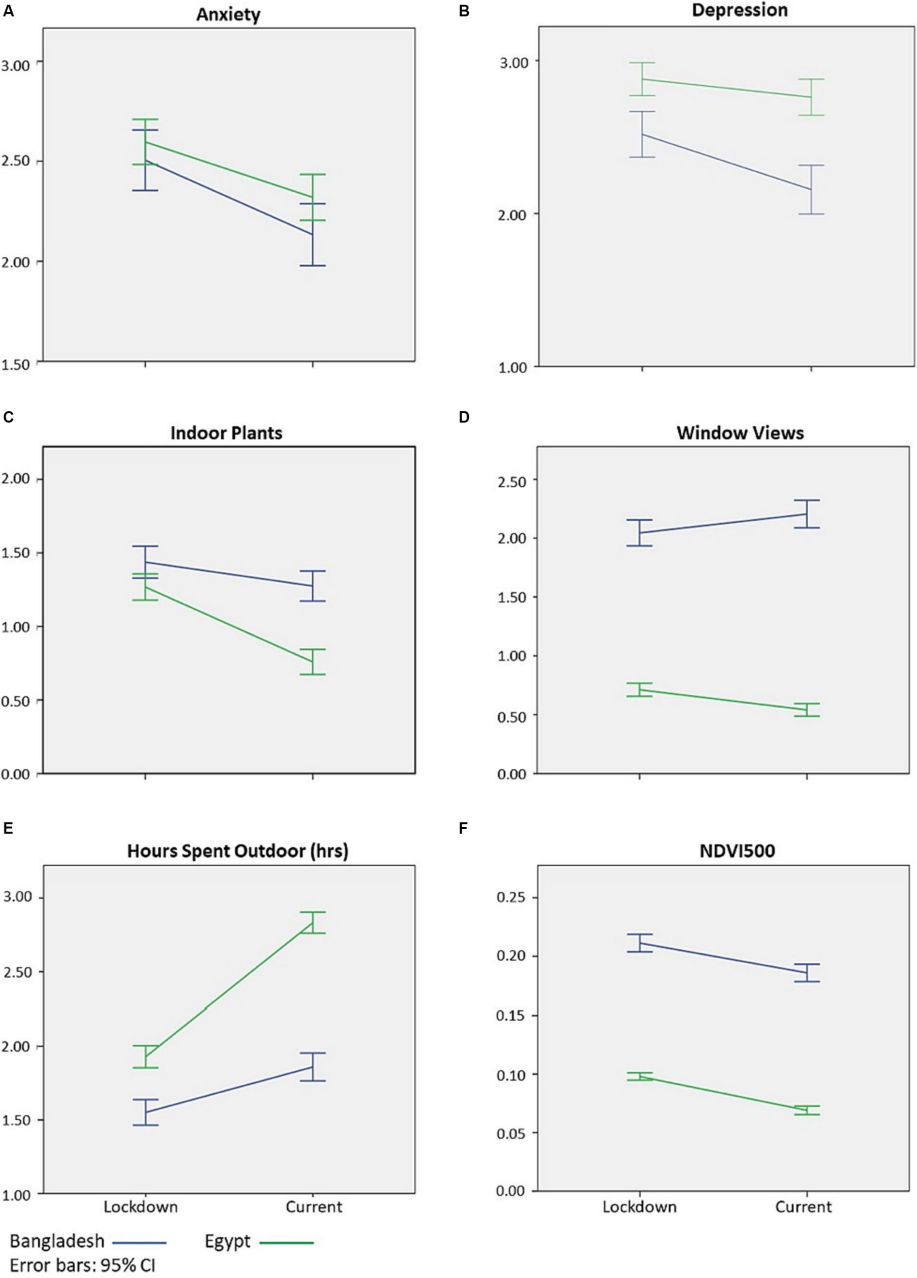
Changes in mental health **(A,B)**, perceived nature exposure **(C–E)**, and greenness **(F)** from the COVID-19 lockdown period to the current time in Bangladesh and Egypt (*N* = 1,216). CI, confidence intervals; NDVI500, normalized difference vegetation index within a 500 m buffer of survey respondent, hrs = hours.

Anxiety and depression levels decreased in Bangladesh and Egypt from the lockdown to the current time (Figure 1, Supplementary Table S1). Bangladesh witnessed more substantial decreases in depression than Egypt. No between-country differences were seen for decreases in anxiety.

Nature access changed from the lockdown to the current time with varying patterns. In both countries, hours spent outdoors increased while indoor plants decreased. Window views of nature increased in Bangladesh but decreased in Egypt. Greenness also increased in Bangladesh but decreased in Egypt.

### 3.3 Associations between changes in mental health and natural exposure

Changes in natural exposure partially corresponded with changes in mental health (Table 2, 3). In Bangladesh, increased outdoor time was associated with decreased anxiety and depression. In Egypt, a increase in the number of indoor plants was associated with increased anxiety but decreased depression. No other changes in natural exposure predicted changes in mental health. Overall, models of nature exposure, socio-demographic variables, potential risk factors, household characteristics, and COVID-19 diagnosis poorly explained changes in anxiety and depression. Variance explained ranged from 3% for depression to 5% for anxiety in Egypt models. The variance explained was greater in Bangladesh models, ranging from 7% for depression to 9% for anxiety.

**Table 2:** Associations between changes in anxiety and nature exposure from the COVID-19 lockdown to the current time in Bangladesh and Egypt (*N* = 1,216).

	Bangladesh (*N* = 556)					Egypt (*N* = 660)				
	*B*	Std. B	95% Cl		*p*	*B*	Std. B	95% Cl		*p*
Gender	0.344	0.095	0.03	0.658	0.032	0.025	0.009	−0.195	0.246	0.822
Age	−0.251	−0.069	−0.562	0.06	0.114	0.022	0.001	−1.343	1.388	0.974
Marital status	−0.342	−0.074	−0.732	0.048	0.085	−0.618	−0.032	−2.419	1.182	0.5
Current place of residence (urban)	0.055	0.013	−0.305	0.416	0.764	0.021	0.005	−0.31	0.352	0.899
Monthly family income	−0.221	−0.135	−0.36	−0.082	0.002	−0.072	−0.026	−0.284	0.14	0.507
COVID-19 diagnosis	−0.135	−0.042	−0.399	0.128	0.313	−0.004	−0.002	−0.16	0.152	0.955
Presence of long-standing illness	−0.47	−0.075	−0.987	0.047	0.075	0.398	0.071	−0.075	0.871	0.099
Habit of smoking	0.506	0.099	0.058	0.953	0.027	0.631	0.072	−0.075	1.338	0.08
BMI	0.024	0.056	−0.012	0.061	0.19	0.022	0.068	−0.003	0.046	0.078
*Nature exposure*										
Δ indoor plants	0.094	0.052	−0.06	0.247	0.232	0.169	0.16	0.088	0.25	0.000
Δ Window views of nature	−0.023	−0.014	−0.157	0.112	0.742	0.072	0.043	−0.056	0.2	0.269
Δ Time spent outdoors	−0.264	−0.156	−0.403	−0.124	0.000	−0.04	−0.037	−0.122	0.042	0.34
Δ NDVI_500_	1.135	0.031	−1.872	4.142	0.459	−1.277	−0.014	−8.271	5.717	0.72
*R*^2^/Adjusted *R*^2^	0.086/0.064					0.046/0.027				

Δ = value at the current time minus value in the lockdown period.

**Table 3:** Associations between changes in depression and nature exposure from the COVID-19 lockdown to the current time in Bangladesh and Egypt (*N* = 1,216).

	Bangladesh (*N* = 556)					Egypt (*N* = 660)				
	*B*	Std. B	95% Cl		*p*	*B*	Std. B	95% Cl		*p*
Gender	0.308	0.083	−0.018	0.633	0.064	0.1	0.031	−0.153	0.352	0.438
Age	−0.285	−0.077	−0.607	0.038	0.084	−0.384	−0.023	−1.945	1.176	0.629
Marital status	−0.203	−0.043	−0.607	0.202	0.326	−0.281	−0.013	−2.34	1.778	0.789
Current place of residence (urban)	0.028	0.006	−0.345	0.402	0.882	0.056	0.011	−0.323	0.434	0.772
Monthly family income	−0.146	−0.087	−0.29	−0.002	0.047	−0.035	−0.011	−0.278	0.207	0.776
COVID-19 diagnosis	−0.045	−0.014	−0.318	0.228	0.747	−0.059	−0.026	−0.237	0.12	0.519
Presence of long-standing illness	0.029	0.004	−0.507	0.565	0.916	0.15	0.024	−0.391	0.692	0.586
Habit of smoking	0.809	0.155	0.345	1.273	0.001	0.474	0.048	−0.334	1.283	0.25
BMI	0.022	0.05	−0.015	0.06	0.242	0.014	0.038	−0.014	0.042	0.336
*Nature exposure*										
Δ indoor plants	0.094	0.051	−0.065	0.253	0.246	−0.119	−0.099	−0.211	−0.026	0.012
Δ Window views of nature	−0.037	−0.023	−0.177	0.102	0.598	−0.138	−0.073	−0.285	0.008	0.063
Δ Time spent outdoors	−0.256	−0.147	−0.4	−0.111	0.001	0.012	0.01	−0.082	0.106	0.801
Δ NDVI_500_	−0.658	−0.017	−3.776	2.459	0.679	−1.223	−0.012	−9.22	6.774	0.764
*R*^2^/Adjusted *R*^2^	0.066/0.044					0.025/0.005				

Δ = value at the current time minus value in the lockdown period.

## 4 Discussion

### 4.1 Summary of main findings

The COVID-19 pandemic had a significant impact on people’s mental health. In LMICs, where access to mental health care is often limited, it is essential to identify alternative strategies to support mental health during these challenging times (17). One such strategy is access to nature, which has numerous mental health benefits (56, 57). However, the extent to which people in LMICs had access to nature and how such exposure influenced mental health recovery across the pandemic remains unclear.

This study investigated changes in nature exposure and associations with changes in mental health in two LMICs. Results showed that mental health improved in Egypt and Bangladesh after lockdowns. In addition, residents of both countries increased their time spent outdoors after lockdowns, and these changes were associated with reductions in anxiety and depression in Bangladesh. Changes in nature exposure were not associated with mental health recovery in Egypt, except that increase in the number of indoor plants were unexpectedly associated with increased anxiety but decreased depression. The study’s focus on mental health recovery aligns with the current global situation of most countries transitioning out of response to recovery. Therefore, the current study’s findings provide insights into how nature exposure might continue to support recovery from poor mental health during the pandemic in LMICs.

The observed decreases in anxiety and depression after lockdowns have been documented in other contexts. For instance, an Italian study showed relief from psychological distress and symptoms after the pandemic (58). The relaxation of lockdown measures may have reduced social isolation, increased social support, and more significant opportunities for engagement in recreational activities (59). Additionally, reducing COVID-19 cases and deaths may have reduced anxiety about the virus and its impact on health (60). While there is a lack of previous studies comparing green space use or access and associations with mental health in LMICs, findings from cross-country studies shed light on the observed differences between Egypt and Bangladesh. Ribeiro et al. (61)examined the relationship between nature exposure and mental health outcomes during the pandemic in Spain and Portugal. They are engaging with natural environments, whether in public or private green spaces, during lockdown periods positively reduced stress and improved mental well-being. Research before the pandemic identified differences between LMICs concerning perceived safety, preferred amenities, and the impact of climate on green space use (62). Considering this body of evidence, it is reasonable to attribute the differences in mental health recovery between Egypt and Bangladesh to various factors, such as variations in the study samples, severity of lockdown measures, cultural approaches to coping mechanisms, and mental health services.

We found that residents in both countries increased their time outdoors after lockdowns, likely due to lockdowns restricting people’s outdoor access. These changes may have led to a greater appreciation of the benefits of spending time in nature and motivated spending time outdoors (63). Similar findings have been seen in Scotland, where 80% of adults visited nature outdoors at least once a week after lockdowns, whereas 71% visited nature outdoors during the initial lockdown period (64). A study in Norway reported that there was a shift from residential and commercial zones toward city green spaces, including forests and protected areas after the lockdown was lifted, indicating a growing interest in nature access among the public during the post-lockdown time (65).

We also found that residents decreased the number of indoor plants after lockdowns, possibly due to shifts in focus from indoor to outdoor spaces. As lockdowns lifted, people may have experienced a greater appreciation for spending time outdoors in natural environments. The restricted mobility and confinement indoors during the lockdown period could have intensified people’s longing for open spaces and green surroundings. This newfound or reinvigorated appreciation for outdoor spaces might have led individuals to prioritize spending time outside rather than investing effort in maintaining indoor plants (66), which may have led to a decrease in their interest in keeping and maintaining indoor plants.

Increased time outdoors after lockdowns being associated with reduced anxiety and depression in Bangladesh aligns with earlier research. Spending time in green space can lead to numerous mental health benefits for mental health, including reduced symptoms of anxiety and depression before the pandemic (56, 57) and during it (38, 67). For instance, Beyer et al. (68) reported that time spent outdoors was negatively associated with depression (68), and Lesser and Nienhuis (69) found that inactive adults who spent more time outdoors during the pandemic experienced greater well-being (69). A study in Austria during the pandemic reported that spending time outdoors was associated with better mental health (70). Collectively, these findings can be explained by the biophilia hypothesis, which posits that humans have an innate connection to nature and that exposure to natural environments can positively impact mental health (71, 72). During lockdowns, people were restricted from going outdoors and connecting with nature. As restrictions lifted, people could spend more time outside and engage in outdoor activities (65). This increased exposure to nature may have contributed to the decrease in anxiety and depression.

Unexpectedly, our study found that increases in indoor plants were associated with increased anxiety and decreased depression in Egypt. Previous research suggests indoor plants positively affect mental health and well-being (28, 73). Residents who spent more time outdoors, engaging in recreational activities, and less time indoors after lockdowns may have felt less worried or panicked (66). In contrast, residents with more indoor plants could have continued indoor pursuits without these specific benefits derived from being outdoors but still felt less depressed while being isolated.

### 4.2 Study limitations

Several limitations to this study should be considered when interpreting the results. First, we used a retrospective study design with a single time point but repeated measures. Retrospective self-reported information on nature exposure and mental health during lockdowns may have been inaccurate. Participants may have underreported access and symptoms due to social desirability bias or other factors. This dataset also precluded us from establishing causal relationships between the variables. Second, we did not measure other potential factors that may have influenced the relationships between nature exposure and mental health. These factors include, but are not limited to, nature connection, types of recreational activities, quality of indoor living spaces, the therapeutic aspects of plant care, and the psychological impacts of isolation. Future studies should consider these elements for a more comprehensive understanding of the multifaceted relationship between nature exposure and mental well-being. Third, we did not explore the mechanisms underlying the relationships between nature access and mental health. Future research could examine the biological, psychological, and social pathways through which natural exposure may influence mental health recovery.

## 5 Conclusion

Residents in two LMICs increased their time outdoors in nature and saw improvements in their mental health after COVID-19 lockdowns. Increases in time outdoors were associated with mental health recovery in one country (Bangladesh). Decreases in the number of indoor plants were associated with contrasting mental health outcomes in the other country (Egypt). Access to outdoor nature exposure, but not necessarily having indoor plants and green window views, may assist with mental health recovery following public health crises. These findings can inform mental health interventions, especially in LMICs and during times of crisis like the COVID-19 pandemic. Encouraging individuals to spend more time outdoors in natural settings could be an effective and accessible strategy for promoting mental well-being. Further, this study underscores the need for further research into the relationship between nature exposure and mental health in LMICs, as well as the importance of incorporating these findings into educational programs for healthcare professionals, urban planners, and the general public.

## Data availability statement

The raw data supporting the conclusions of this article will be made available by the authors, without undue reservation.

## Ethics statement

The studies involving humans were approved by ethics committee of the Institute of Disaster Management, Khulna University of Engineering and Technology, Khulna, Bangladesh and Psychology Department, Faculty of Arts, Menoufia University, Egypt. The studies were conducted in accordance with the local legislation and institutional requirements. The participants provided their written informed consent to participate in this study.

## Author contributions

MP: Conceptualization, Data curation, Formal analysis, Investigation, Methodology, Writing – original draft, Writing – review & editing. MoB: Conceptualization, Formal analysis, Investigation, Methodology, Writing – original draft, Writing – review & editing. Hİ: Conceptualization, Formal analysis, Investigation, Methodology, Writing – original draft, Writing – review & editing. MaB: Conceptualization, Formal analysis, Investigation, Writing – review & editing. ASD: Conceptualization, Formal analysis, Investigation, Methodology, Writing – original draft, Writing – review & editing. MHa: Formal analysis, Methodology, Writing – review & editing. MHe: Formal analysis, Methodology, Writing – review & editing. SA: Formal analysis, Investigation, Methodology, Writing – review & editing. AMD: Formal analysis, Investigation, Methodology, Writing – review & editing. FS: Formal analysis, Investigation, Methodology, Writing – review & editing. MAl: Formal analysis, Methodology, Writing – review & editing. SB: Formal analysis, Funding acquisition, Investigation, Methodology, Writing – review & editing. MK: Formal analysis, Funding acquisition, Investigation, Writing – review & editing. MHo: Data curation, Formal analysis, Investigation, Writing – review & editing. MAz: Formal analysis, Methodology, Writing – review & editing. MR: Data curation, Investigation, Methodology, Writing – review & editing. SS: Formal analysis, Methodology, Writing – review & editing. RS: Formal analysis, Methodology, Writing – review & editing. JM-I: Investigation, Methodology, Writing – review & editing. DB-A: Investigation, Methodology, Writing – review & editing. AR-M: Formal analysis, Investigation, Methodology, Writing – review & editing.

## Footnotes

^1^https://www.kobotoolbox.org/
