# Supplementary material for: Exposure to urban green spaces and mental health during the COVID-19 pandemic: evidence from two low and lower-middle-income countries
Source: Front Public Health. 2024 Mar 1;12:1334425. doi: 10.3389/fpubh.2024.1334425 (PMC10940342; doi:10.3389/fpubh.2024.1334425)
Supplement: Supplementary file 1 [file Data_Sheet_1.docx]

**Supplemental material**

**
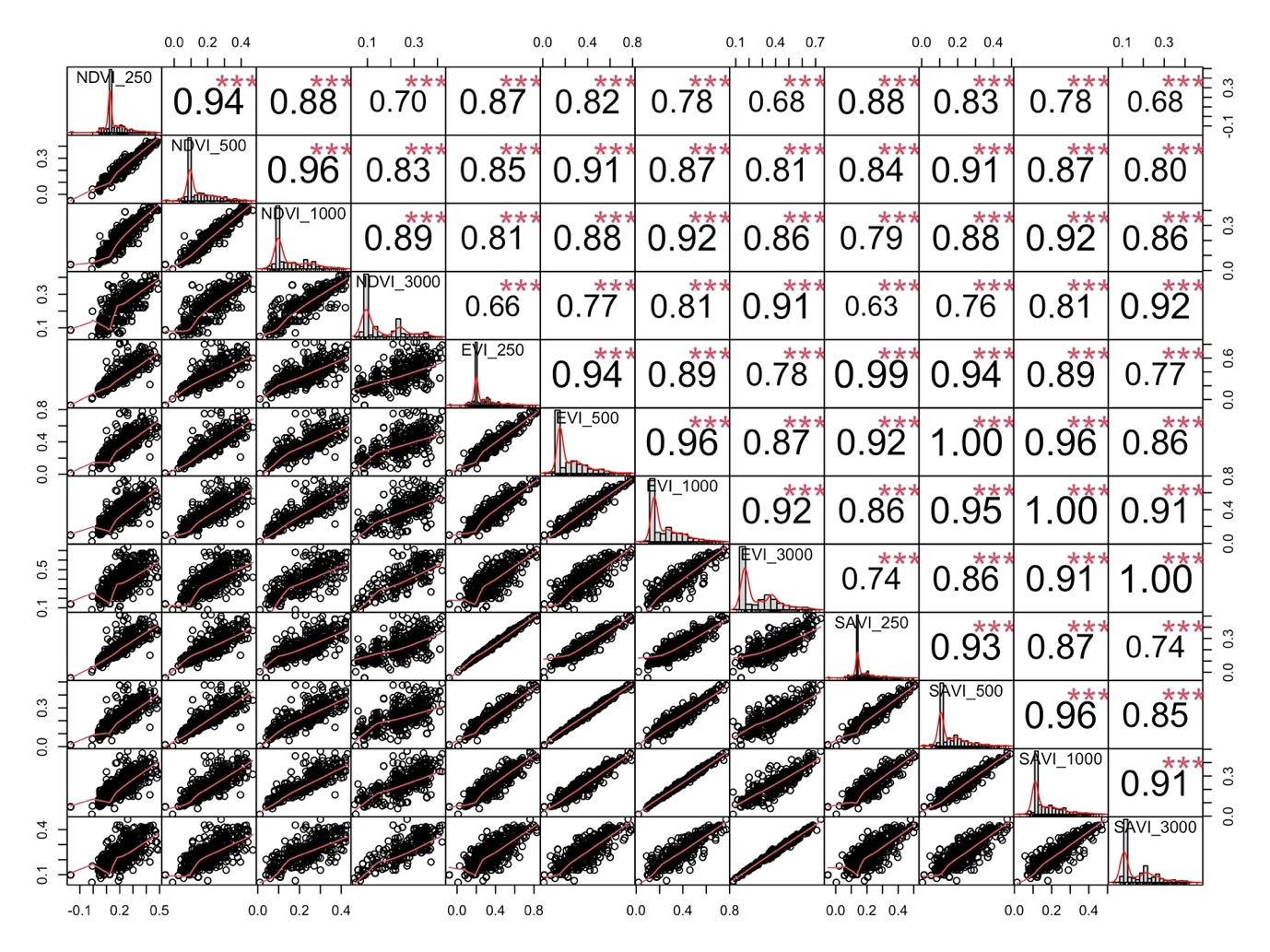
**

**Figure S1.** Correlations between estimates of greenness including NDVI 250, NDVI 500, NDVI 1000, NDVI 3000, EVI 250, EVI 500, EVI 1000, EVI 3000, SAVI 250, SAVI 500, SAVI 1000, SAVI 3000

**Table S1.** Changes in mental health, perceived nature exposure, and greenness in Bangladesh and Egypt during lockdown vs. the current time (N = 1216).

|  | **Bangladesh (N=556)** | | | | **Egypt (N=660)** | | | | **Country Differences (N=1216)** | | | |
| --- | --- | --- | --- | --- | --- | --- | --- | --- | --- | --- | --- | --- |
|  | Lockdown | Current | Difference^a^ | | Lockdown | Current | Difference^a^ | | **Δ**Bangladesh | **Δ**Egypt | Difference^a^ | |
|  |  |  | *t* | *p* |  |  | *t* | *p* |  |  | *t* | *p* |
| **Mental health** | |  |  |  |  |  |  |  |  |  |  |  |
| Anxiety | 2.5 | 2.13 | -4.86 | <.000 | 2.6 | 2.31 | -5.44 | <.000 | -0.37 | -0.28 | -1.06 | .29 |
| Depression | 2.52 | 2.15 | -4.60 | <.000 | 2.89 | 2.76 | -2.05 | .041 | -0.36 | -0.12 | -2.55 | .011 |
| **Perceived exposure** | |  |  |  |  |  |  |  |  |  |  |  |
| Indoor plants | 1.437 | 1.275 | -3.83 | 0.000 | 1.27 | 0.76 | -10.57 | 0.000 | -0.16 | -0.51 | 5.32 | 0.000 |
| Window views | 2.04 | 2.2 | 6.81 | 0.001 | 0.71 | 0.54 | 17.91 | 0.000 | 0.16 | -0.17 | 5.96 | 0.000 |
| Hours spent outdoors | 1.55 | 1.86 | 3.29 | 0.000 | 1.92 | 2.77 | -5.62 | 0.000 | 0.305 | 0.85 | 8.209 | 0.000 |
| **Greenness** |  |  |  |  |  |  |  |  |  |  |  |  |
| NDVI_500_ | 0.21 | 0.19 | 12.23 | 0.000 | 0.07 | 0.1 | -48.49 | 0.000 | -0.02 | -0.03 | 1.721 | 0.85 |

Notes: ^a^t-test comparing lockdown and current means

**Table S2.** VIF Scores

|  | **Bangladesh** | | **Egypt** | |
| --- | --- | --- | --- | --- |
|  | Anxiety | Depression | Anxiety | Depression |
| Gender | 1.164 | 1.164 | 1.033 | 1.033 |
| Age | 1.133 | 1.133 | 1.439 | 1.439 |
| Marital status | 1.099 | 1.099 | 1.507 | 1.507 |
| Current place of residence (urban) | 1.051 | 1.051 | 1.041 | 1.041 |
| Monthly family income | 1.111 | 1.111 | 1.043 | 1.043 |
| COVID-19 diagnosis | 1.029 | 1.029 | 1.064 | 1.064 |
| Presence of long-standing illness | 1.034 | 1.034 | 1.249 | 1.249 |
| Habit of smoking | 1.188 | 1.188 | 1.14 | 1.14 |
| BMI | 1.067 | 1.067 | 1.012 | 1.012 |
| Δ indoor plants | 1.105 | 1.105 | 1.033 | 1.033 |
| Δ window views of nature | 1.12 | 1.12 | 1.027 | 1.027 |
| Δ time spent outdoors | 1.038 | 1.038 | 1.022 | 1.022 |
| Δ NDVI_500_ | 1.028 | 1.028 | 1.026 | 1.026 |

**Table S3.** Skewness and Kurtosis Scores

|  | Bangladesh | | Egypt | |
| --- | --- | --- | --- | --- |
|  | Anxiety | Depression | Anxiety | Depression |
| Skewness | -0.237 | -0.073 | 0.323 | -0.128 |
| Kurtosis | 0.874 | 0.957 | 2.199 | 0.683 |

Table S4. Cronbach’s Alpha Scores

|  | Bangladesh | | Egypt | |
| --- | --- | --- | --- | --- |
|  | Lockdown | Current | Lockdown | Current |
| Depression | 0.552 | 0.658 | 0.684 | 0.84 |
| Anxiety | 0.582 | 0.658 | 0.617 | 0.669 |
